# Dual Fixation of Calcaneal Tuberosity Avulsion with Concomitant Achilles Tendon Rupture: A Novel Hybrid Technique

**DOI:** 10.1155/2017/9150538

**Published:** 2017-03-05

**Authors:** Gautham Prabhakar, Nicholas Kusnezov, Nicholas Rensing, Amr Abdelgawad

**Affiliations:** ^1^Paul L. Foster School of Medicine, Texas Tech Health Sciences Center El Paso, El Paso, TX, USA; ^2^William Beaumont Army Medical Center, El Paso, TX, USA

## Abstract

Fracture of the calcaneal tuberosity with a concomitant Achilles tendon rupture presents a difficult challenge for the treating surgeon. The ultimate goal of treatment is to restore function of both the gastrocnemius-soleus complex and the Achilles tendon. This particular subset of fractures occurs often in diabetics and elderly patients with osteoporosis making fixation of the displaced fragment rather complex. If the Achilles tendon disruption is only discovered later once the fracture is healed, subsequent management is difficult with surgical treatment being more morbid. While this is a rare injury, the consequences of a missed chronic Achilles tendon disruption are severe with significant dysfunction. It is therefore important to have a high index of suspicion for concomitant injury and to be prepared for dual fixation. We present a novel hybrid surgical fixation technique, which may be used in this instance.

## 1. Introduction

Calcaneal tuberosity avulsions are relatively rare, constituting 1–3% of all calcaneal fractures [1–3]. Increasing age, diabetes mellitus, and reduced bone mineral density portend an increased risk of tuberosity avulsion [4, 5]. These injuries are most often sustained through forced dorsiflexion of a maximally plantar-flexed foot [6]. The Achilles tendon typically fails by the same mechanism [7], though there are no reports to date of simultaneous tuberosity avulsion and complete Achilles tendon rupture. We present a case of a displaced calcaneal tuberosity avulsion with concomitant Achilles tendon rupture necessitating repair at the time of fracture fixation and discuss a novel hybrid technique of dual fixation that helps to increase the stability of the fracture fixation and alleviate the stresses exerted by the pull of the Achilles tendon.

## 2. Case Presentation

A 58-year-old diabetic female presented to the emergency department with posterior heel pain and inability to ambulate following a misplaced step into a hole the previous night. She reported forced dorsiflexion of the ankle and otherwise denied any prodromal symptoms. Her medical history was significant for well-controlled type 2 diabetes mellitus (with a most recent hemoglobin A1C of 6.3) and smoking.

Physical examination of her posterior heel demonstrated intact but attenuated skin with early soft tissue necrosis. Additionally there was a palpable gap over her heel with crepitance. The patient was unable to move the ankle due to pain.

**Figure 1:**
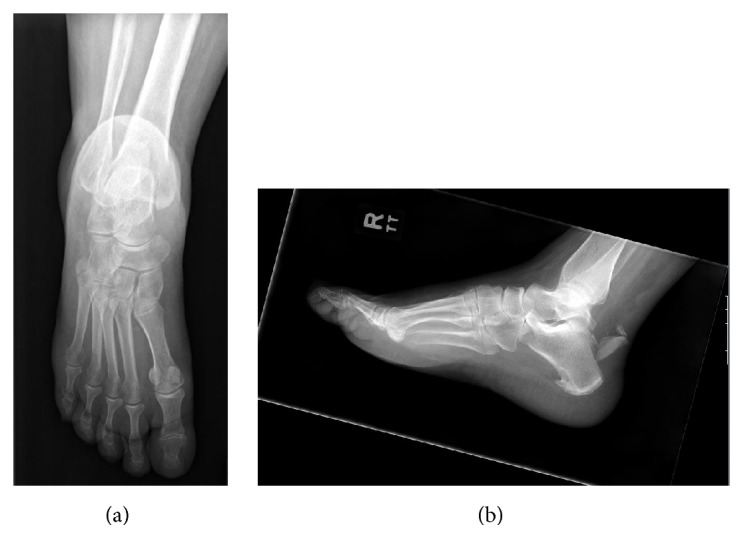
Anteroposterior (a) and lateral (b) radiographs demonstrating displaced fragmented calcaneal tuberosity avulsion following injury.

Injury radiographs were significant for a displaced fragmented calcaneal tuberosity avulsion (Figures 1(a) and 1(b)). Given the impending soft tissue compromise, the patient was taken urgently for fracture reduction and internal fixation. Surgery was performed approximately 3 to 4 hours after her presentation to the emergency department.

Percutaneous reduction was initially attempted given the patient's comorbidities and resultant increased risk of wound complications, but this ultimately proved inadequate and necessitated conversion to open reduction through a posterolateral approach. An incision was made lateral to the area of compromised skin in an attempt to mitigate wound complications.

**Figure 2:**
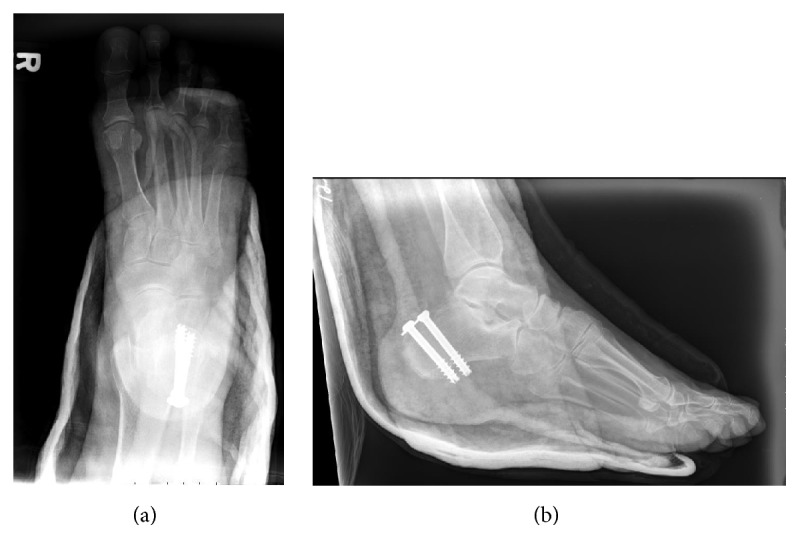
Anteroposterior (a) and lateral (b) radiographs taken immediately postoperatively.

Intraoperatively, the Achilles tendon was found to be partially attached to the largest avulsed calcaneal tuberosity fragment. The remaining portion of the tendon, constituting approximately 50%, was avulsed off the tuberosity without a sizable bony fragment. The main tuberosity fragment was first reduced with a large two-point tenaculum clamp and fixed with two cannulated 7.3 mm screws from posterosuperior to anteroinferior. Attention was then given to the ruptured Achilles tendon. The proximal stump was secured with #2 FiberWire (Arthex) suture via Krackow technique. The suture was then passed by free straight Keith needle through the cannulated screws, exiting plantarly through the heel pad where the two tails were tied over a padded button (Figures 2(a) and 2(b)). This technique has been previously described for tendon fixation in the foot and ankle [8]. The fixation was felt to be excellent and the patient was immobilized in a posterior slab splint in 30 degrees of equinus to protect the repair.

**Figure 3:**
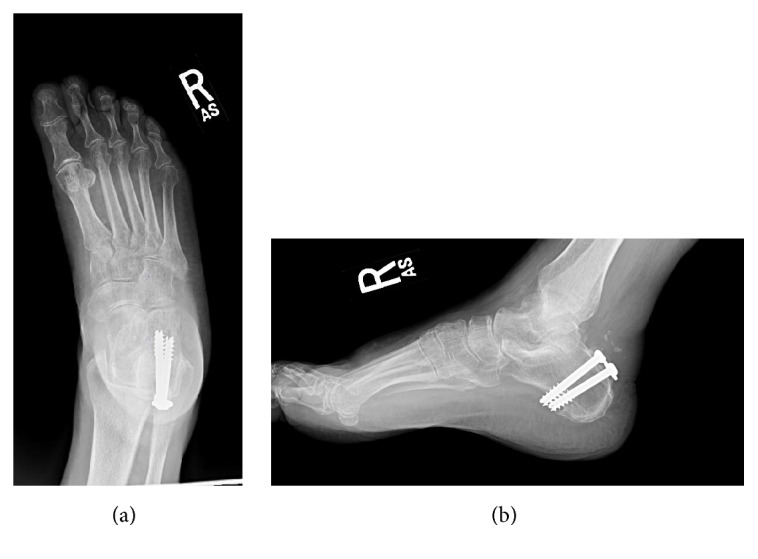
Anteroposterior (a) and lateral (b) radiographs showing complete healing of the tuberosity.

Postoperatively, the patient was immobilized and made non-weight-bearing with a walker for the 6 weeks. She was seen back in aftercare at the 2-week, 6-week, and 3-month intervals. Weight-bearing was advanced to partial and finally to full at 12 weeks. The necrotic soft tissue resolved with local wound care by the 6-week visit. The suture was cut at 6 weeks and the button removed. Radiologic union of the fracture fragments was evident at 6 weeks. At her latest 6-month follow-up, she had returned to full weight-bearing without pain and full range of motion and restored symmetric plantar flexion strength. She had clinically full-strength triceps surae function and could stand on her tiptoes. Final radiographs demonstrated complete healing of the tuberosity (Figures 3(a) and 3(b)).

## 3. Discussion

The optimal management of calcaneal fractures remains a subject of debate and continues to evolve. Beavis and colleagues [9] describe a modified classification for calcaneal tuberosity avulsions. In this system, a type I fracture refers to a “sleeve” type or true avulsion fracture, type II is the “beak” fracture, type III is an infrabursal avulsion from the middle third of the posterior tuberosity, and finally type IV is a variation of the beak where a small triangular fragment is separated from the upper border of the tuberosity [7, 10–12]. It must be noted that some beak fractures can receive fibers from the Achilles tendon [10, 11]. Rijal et al. [13] reported a case of a multifragmentary tuberosity avulsion in which the Achilles was not in continuity with the smaller of the two fragments but did not necessitate repair. However, in our case there was a complete Achilles tendon disruption with half the tendon attached to the fracture fragment and the other portion avulsed off the tuberosity. This is a rather unusual variant that does not clearly fall into either of the calcaneal tuberosity avulsion types. Given the significant deforming force of the triceps surae on the avulsed tuberosity fragment and the thin soft tissue envelope of the posterior heel, these injuries are universally operative [14].

There are a wide variety of surgical techniques proposed for calcaneal tuberosity fractures. Lag screw fixation is among the most common techniques described for tuberosity fixation [9, 15, 16]. However, lag screw fixation is only appropriate in the setting of large tuberosity fragments with good bone quality, which is rarely the case. Screw fixation has furthermore been associated with iatrogenic comminution of thin avulsion fragments as well as hardware prominence causing posterior skin compromise [16].

Tension band constructs have also been suggested [4]. Squires et al. [4] illustrated a technique in which the avulsed tuberosity fragment is reduced followed by fixation with two K-wires placed from superior and posterior to inferior and anterior. A figure-8 tension band wire is then passed around the ends of the K-wire over the lateral wall of the calcaneus. Despite the fact that the tension band adequately neutralizes the force of the Achilles tendon, a bulky construct on the posterior or lateral aspect of the calcaneus is necessitated, which may lead to peroneal tendon irritation and/or soft tissue complications [17].

Suture anchors have been utilized for the treatment of avulsion injuries with minimal bony involvement [5]. Janis et al. [18] proposed that soft tissue anchors may be a better option for fixation of tendon to the calcaneus in the surgical management of Achilles tendon ruptures, as screw fixation alone is not effective in resisting the massive pull out tension of the triceps surae. In this technique, the author does not specify if the bony fragment is excised or incorporated into the repair. In a recent study, Yoshida et al. [19] suggested that the soft anchor bridge technique with screws provides increased fixation strength compared with the use of screws or anchors alone. The supplemental suture bridge utilized in the present report may similarly serve as a more favorable environment to promote bony and tendinous healing.

To the authors' knowledge, this is the first case describing a hybrid fixation construct for the unique case of combined calcaneal tuberosity avulsion and complete Achilles tendon disruption. Banerjee et al. [20] describe a technique, which is similar to ours in which the small avulsed tuberosity fragment and Achilles tendon are affixed with a modified Krackow suture. The tails are then passed plantigrade through bone tunnels drilled in the body of the calcaneus and tied through a small incision on the plantar aspect of the heel. This technique is advantageous in that it can be used independently for smaller fracture fragments. Our tuberosity fragment was substantial and required additional internal fixation. In our case, we used a similar technique for the Achilles tendon rupture; however sutures were passed through the cannulated screws exiting plantarly through the heel pad where the two tails were tied over a padded button. This is a described technique with which significant complications of wound infection or pressure necrosis have not been associated [8]. Furthermore, the added screw fixation provides the advantage of stabilizing the large tuberosity piece. While our patient went on to heal uneventfully, careful attention should be paid to the button postoperatively to ensure no signs of pressure necrosis are evident, especially in patients with diminished plantar sensation.

Fortunately, given the expedient fixation in this case and avoidance of the compromised soft tissue with a posterolateral approach, our patient experienced no major adverse sequelae from the soft tissue necrosis despite her late presentation and her comorbidities. Although open repair may increase the risk of postoperative wound complications [21], this method may be a better alternative to doing it percutaneously. The merit to this is that the Achilles tendon disruption could go unnoticed if the tuberosity fracture was fixed percutaneously. If the Achilles tendon disruption is only discovered later once the fracture is healed, subsequent management would have been difficult with surgical treatment being more morbid [22].

While this is a rare injury, the consequences of a missed chronic Achilles tendon disruption are severe with significant dysfunction. It is therefore important to have a high index of suspicion for concomitant injury and to be prepared for dual fixation. While MRI would certainly yield additional information on the integrity of the tendon in relation to each fragment, this may be impractical given the cost, delay in surgery, and rarity of the injury. We cannot recommend open treatment of all tuberosity avulsions; however, we believe that percutaneous approaches risk missing this type of injury, especially in the case of a multifragmentary avulsion. We present a novel hybrid fixation technique, which may be used in this instance. The technique provided excellent stability for both the tendon repair and fracture fixation alleviating the stresses created by the Achilles tendon pulling the avulsed segment.
